# Heparan sulfate targeting strategy for enhancing liposomal drug accumulation and facilitating deep distribution in tumors

**DOI:** 10.1080/10717544.2020.1745326

**Published:** 2020-04-03

**Authors:** Ping-Hsueh Kuo, Yi-Hsien Teng, Ann-Lun Cin, Wen Han, Pei-Wan Huang, Lily Hui-Ching Wang, Yu-Ting Chou, Jia-Ling Yang, Yun-Long Tseng, Minhsiung Kao, Margaret Dah-Tsyr Chang

**Affiliations:** a Institute of Molecular and Cellular Biology, National Tsing Hua University, Hsinchu, Taiwan;; b Operations Center for Industry Collaboration, National Tsing Hua University, Hsinchu, Taiwan;; c Graduate Program of Biotechnology in Medicine, National Tsing Hua University, Hsinchu, Taiwan;; d Mackay Memorial Hospital, Hsinchu, Taiwan;; e Institute of Biotechnology, National Tsing Hua University, Hsinchu, Taiwan;; f Taiwan Liposome Company, Ltd, Taipei, Taiwan;; g Department of Life Science, National Tsing Hua University, Hsinchu, Taiwan

**Keywords:** Desmoplastic stroma, extracellular matrix, heparan sulfate, tumor penetration, drug delivery

## Abstract

Nanoparticles (NPs), such as liposomes, effectively evade the severe toxicity of unexpected accumulation and passively shuttle drugs into tumor tissues by enhanced permeability and retention. In the case of non-small cell lung cancer and pancreatic ductal adenocarcinoma, cancer-associated fibroblasts promote the aggregation of a gel-like extracellular matrix that forms a physical barrier in the desmoplastic stroma of the tumor. These stroma are composed of protein networks and glycosaminoglycans (GAGs) that greatly compromise tumor-penetrating performance, leading to insufficient extravasation and tissue penetration of NPs. Moreover, the presence of heparan sulfate (HS) and related proteoglycans on the cell surface and tumor extracellular matrix may serve as molecular targets for NP-mediated drug delivery. Here, a GAG-binding peptide (GBP) with high affinity for HS and high cell-penetrating activity was used to develop an HS-targeting delivery system. Specifically, liposomal doxorubicin (L-DOX) was modified by post-insertion with the GBP. We show that the *in vitro* uptake of L-DOX in A549 lung adenocarcinoma cells increased by GBP modification. Cellular uptake of GBP-modified L-DOX (L-DOX-GBP) was diminished in the presence of extracellular HS but not in the presence of other GAGs, indicating that the interaction with HS is critical for the cell surface binding of L-DOX-GBP. The cytotoxicity of doxorubicin positively correlated with the molecular composition of GBP. Moreover, GBP modification improved the *in vivo* distribution and anticancer efficiency of L-DOX, with enhanced desmoplastic targeting and extensive distribution. Taken together, GBP modification may greatly improve the tissue distribution and delivery efficiency of NPs against HS-abundant desmoplastic stroma-associated neoplasm.

## Introduction

Nanoparticle (NP)-based drugs such as liposomes, polymeric NPs, and micelles effectively avoid severe side effects of unexpected drug aggregation by shuttling drugs into tumor tissues through the enhanced permeability and retention (EPR) effect (Matsumura & Maeda, 1986; Greish, 2010). However, the gel-like fibrotic tumor extracellular matrix (ECM), which is composed of a protein network containing collagen and elastic fibers and proteoglycans, especially glycosaminoglycans (GAGs), greatly compromises tumor-penetrating performance, leading to insufficient extravasation and limited efficiency for NP therapy (Lee et al., 2010; Li et al., 2012; Nishihara, 2014; Yang & Gao, 2017). Aberrantly high expression of ECM elicited by cancer-associated fibroblasts (CAFs) constitutes compact physical barriers in desmoplastic stroma to restrain chemical and NP therapies from interstitial transport in multiple solid tumor systems, including non-small cell lung cancer (NSCLC) (Bremnes et al., 2011), pancreatic ductal adenocarcinoma (Tjomsland et al., 2011), aggressive urothelial carcinoma (Cheng et al., 2009), and breast cancer (Yamashita et al., 2012). Therefore, the development of a strategy to overcome the stroma barrier and to reduce off-target NP localization has emerged as a potential therapeutic approach.

The desmoplastic stroma of a malignant tumor constitutes a tumor microenvironment that consists of closely interacting elements, including stromal cells, endothelial cells, cytokines, and ECM (Maeshima et al., 2002; Tjomsland et al., 2011; Murakami et al., 2018). The desmoplastic stroma provides structural and functional support in the cell growth of normal tissues and tumor nodules and protects cancer cells from immune cell attack and most therapeutic agents (Hodkinson et al., 2007; Keeratichamroen et al., 2018; Lee et al., 2018). Inside the tumor microenvironment, heparan sulfate (HS), one of the highly negatively charged sulfated GAGs, and related HS proteoglycans, HSPGs, mediate the activation of chemokines, enzymes and growth factors involved in cell-matrix interactions (Marolla et al., 2015; Rangel et al., 2018). During tumorigenesis, HS and HSPG are often overexpressed on the cell surface and accumulate on the tumor ECM in secreted and shedded forms, implicating that high-level HSPGs in tumor ECM contribute significantly to tumor progression (Naba et al., 2012; Kawahara et al., 2014). Alteration of HSPG expression in the tumor microenvironment may result in structural and functional consequences, thus influencing tumor progression. In addition, either surface-bound HS/HSPG tethered as part of the endothelial glycocalyx or secreted HSPG that spreads in the perivascular matrix is essential for triggering tumor angiogenesis (Fuster & Wang, 2010). Unlike other ECM components, HS chains can facilitate diffusion of ligands by allowing them to bind and slide or dissociate/reassociate through adjacent binding sites, which has the potential to control the movements of the HS binding factor between communicating cells (Sarrazin et al., 2011). Moreover, HS also works as a critical internalization receptor through the endocytic route for cargos such as biomacromolecules and NPs (Payne et al., 2007; Christianson & Belting, 2014).

Cell-penetrating peptides (CPPs) commonly possess a high content of positively charged residues, including arginine and lysine, bind to sulfated GAGs or phospholipids on the cell surface and translocate through the membrane *via* endocytosis or direct translocation (Ter-Avetisyan et al., 2009; Koren & Torchilin, 2012). CPP shuttles cargos through the plasma membrane and possesses excellent internalization activity *in vitro*. However, most CPPs have low stability and a lack of selectivity for targeting cells *in vitro* and *in vivo* (Huang et al., 2013). Recently, several studies suggested that hydrophobic residues, such as tryptophan (Trp), within the peptide structure synergistically enhance the penetration potential of arginine-rich CPP (Rydberg et al., 2012; Jobin et al., 2015). Moreover, Trp-containing CPP specifically interacts with GAGs, especially HS, in which either the Arg/Lys residue comes into contact with the sugar units by electronic and hydrogen bond interactions with the sulfates or Trp residue mediated by hydrophobic interactions to the sugar rings or sulfate groups of GAGs through π–anion interactions (Bechara et al., 2013). This phenomenon might contribute to selectivity for cancer cells (Jobin et al., 2013). To avoid off-target accumulation and enhance penetration of NPs *in vivo*, a Trp-containing CPP, named GAG-binding peptide (GBP, NYRWRCKNQN), was designed with a specific binding affinity to HS (K*_d_*: 0.70 µM to HS). GBP contains high cell-penetrating activity and exhibits unique molecular interactions with cell surface HSPG; thus, GBP was adopted in an HS-targeting delivery strategy (Fang et al., 2013). GBP binds negatively charged molecules, including HS and lipids, on the cell surface and targets HS-rich tissues, such as mucosal and intestinal tissues as well as epithelial tumor tissues *in vivo* (Fang et al., 2013; Lien et al., 2013; Chen et al., 2015). Unlike other CPPs, GBP recognizes heparin by 2 charged residues, Arg^5^ and Lys^7^, and inserts the membrane by an irreplaceable moiety of an aromatic residue, Trp^4^. Both of these residues coordinately interact with HS and the lipid membrane on the cell surface, contributing to the induction of epithelial cell uptake by macropinocytosis (Hung et al., 2017). In this study, the Cys^6^ residue of GBP was utilized to covalently conjugate with lipids to insert into liposomes. Cys^6^ is not involved in HS and membrane binding properties, and replacement with serine resulted in over 70% cell-penetrating and cargo delivery activities *in vitro* (Fang et al., 2013; Hung et al., 2017). Consumption of the Cys^6^ residue efficiently avoids GBP dimerization.

In this study, we describe an HS-targeting strategy to facilitate liposomes to localize endothelium, distribute deeply into tumor tissues, and induce endocytic cellular uptake by a Trp-containing CPP modification. The ECM is often more abundant and extensive than receptors that express on the tumor cell surface and is accessible from the bloodstream. We hypothesized that targeting HS might serve as a potential strategy for NP-mediated therapy against desmoplastic stroma-associated neoplasms. Human lung adenocarcinoma (ADC), A549 cell line, was used as a desmoplastic tumor model to analyze the HS-targeting strategy of NP. A549 cells were reported with high level HS expression and consecutive shedding of HSPGs in previous studies, and A549 cells and fibroblasts cooperated to facilitate ECM accumulation *in vitro* and in xenograft model (Supplementary Figure S1) (Berry et al., 1991; Hayashida et al., 2008; Nam & Park, 2012; Scherzer et al., 2015; Keeratichamroen et al., 2018). Herein, liposomal doxorubicin (L-DOX) with surface modification by GBP was generated to evaluate a novel strategy to overcome the biological barriers in the lung ADC model. Moreover, a comparison of therapeutic effects between unmodified L-DOX and different formulated GBP-modified L-DOX (L-DOX-GBP) treatments was performed. In summary, the HS-targeting strategy successfully improves both drug accumulation in tumor tissues and tumor distribution of L-DOX, contributing to strong anticancer efficacy *in vivo.* Our engineering strategy may provide an alternative solution to address unmet medical needs by enhancing the therapeutic efficiency of liposomal drugs and alleviating chemotherapeutic side effects in cancer therapy.

## Materials and methods

### Reagents

Doxorubicin hydrochloride and idarubicin hydrochloride were separately purchased from Sigma-Aldrich, Inc. (Germany) and AdooQ bioscience, LLC. GBP peptide (NYRWRCKNQN) was synthesized by Kelowna International Scientific Inc. (Taiwan). Distearoyl phosphatidyl ethanolamine (DSPE)-polyethylene glycol–maleimide (DSPE-PEG_2k_-mal)(SUNBRIGHT^®^DSPE-020MA) was purchased from NOF America Corporation (US), and methoxyl PEG DSPE, Mw2000 (DSPE-mPEG_2k_) was purchased from NANOCS (US). High molecular weight heparin (HMWH), chondroitin sulfate type B (CSB), hyaluronic acid (HA) were purchased from Sigma-Aldrich, Inc., and low molecular weight heparin (LMWH) were purchased from Iduron (UK). AlamarBlue^®^ reagent was purchased from Bio-Rad (US). Rat anti-mouse CD31 antibody (Clone 390) was purchased from eBioscience Inc, and Alexa Fluor^®^ 594 or Fluor^®^ 488 labeled-goat anti-rat IgG or anti-mouse IgG antibodies were purchased from Jackson ImmunoResearch Inc. (US).

### Cell lines

A549, human ADC cell line, was obtained from Bioresource Collection and Research Center (BCRC), Taiwan. A549-near-infrared fluorescent protein (A549-iRFP) cell line was stably expressed near-infrared fluorescent protein (Filonov et al., 2011). NIH-3T3, mouse fibroblast cell line, was obtained from ATCC. A549 and NIH-3T3 cells were respectively cultured in RPMI-1640 medium (Corning) and DMEM (Corning) containing 10% fetal bovine serum (Biological Industries, USA) and 1% PSA (Penicillin, Streptomycine, and Amphotericin) (Gibco, USA) at 37 °C in a humidified atmosphere containing 5% CO_2_.

### Preparation and purification of DSPE-PEG_2k_-GBP

DSPE-PEG_2k_-GBP was synthesized by modifying a method previously reported (Rivest et al., 2007). DSPE-PEG_2k_-mal and GBP at molar ratio 1:1.1 were gently mixed into a pH 8 buffer solution containing 1 mM EDTA, 50 mM trisodium phosphate hydrate, 150 mM sodium chloride, and 50 mM triethanolamine, followed by stirring at 4 °C for 16 h. The buffer of synthesized product was replaced with deionized water using dialysis membrane (Spectra/Por^®^, MWCO 2 kDa), and lyophilized for followed purification.

### Apparatus and chromatographic conditions

Reversed-phase high performance liquid chromatography (HPLC) purification and measurements were carried out using the 2796 Bioseparations Module (Water, USA) and a Photodiode Array detector. Separation was performed on XBridge C18 HPLC column (250 mm × 4.6 mm, particle size 5 μm, Waters). Mobile phase was methanol (20% to 100%), and flow rate, temperature, wavelength were respectively set at 1 ml/min, 55 °C, and 270–280 nm for DSPE-PEG_2k_-GBP.

### Preparation of GBP-modified liposome

The post-insertion method has been widely used for the incorporation of liposomes with phospholipid-modified peptides (Allen et al., 2002). PEGylated L-DOX (HSPC:Chol:DSPE-mPEG_2K_=3:2:0.045) and DiI-loaded liposome (L-DIL) (HSPC:Chol:DSPE-mPEG_2K_=3:2:0.045) 100–120 nm in diameter were kindly provided by Taiwan Liposome Company, Ltd. (Taiwan). Drug encapsulation efficacy (EE) and drug loading capacity (LC) were 98.5% and 5.8%, respectively. Liposome and DSPE-PEG_2K_-GBP (1, 2, 3 mol% of total phospholipid) or DSPE-mPEG_2K_ (3 mol% of total phospholipid) were separately mixed and incubated at 60 °C for 30 min. Subsequently, the mixture was immediately cooled on ice for 15 min and stored in PBS at 4 °C, and the new formulations of L-DOX (or L-DIL) were generated with different density levels of GBP (1 mol%, 2 mol% and 3 mol% labeled as L-DOX-GBP_(L)_, L-DOX-GBP_(M)_, and L-DOX-GBP_(H)_, respectively).

### Characterization of particle size and zeta potential

Particle size and zeta potential of liposome in 9.4% sucrose solution were separately measured by Zetasizer, Nano ZS (Malvern, UK).

### Evaluation of cellular uptake

Quantification of cellular uptake of doxorubicin was analyzed by flow cytometry (AccuriTM C6 Cytometer, BD). Briefly, A549 cells were incubated with different formulations of L-DOX at 37 °C for 24 h. After incubation, the cells were detached, centrifuged at 4 °C, and washed by ice-cold PBS. Finally, the cells were resuspended and analyzed by flow cytometry. The data are based on the mean fluorescence signal for 10,000 cells collected.

### Cytotoxicity analysis

A549 cells were incubated with different formulations of L-DOX at indicated concentration at 37 °C for 24 h, followed with refreshed serum free-medium and incubated to 48 h. After incubation, alamarBlue^®^ cell viability assay was performed. The absorbance at 570 nm and 600 nm wavelength was measured by ELISA reader (800 TS absorbance reader, BioTek).

### GAG competition effect

Three types of GAG including heparan sulfate (HMWH and LMWH), CSB, and HA were used to block GBP-mediated L-DOX uptake. Cells were respectively co-incubated with L-DOX-GBP_(H)_ (10 μM) as well as indicated concentration of GAG at 37 °C for 24 h, and cell uptake of 10 μM L-DOX was set as control to normalize the uptake upon GAG competition.

### Evaluation of nanoparticle penetration activity in heterospheroids

Stroma-rich 3D heterospheroids were established to mimic the tumor nets surrounded by fibroblasts and high-density ECM (Priwitaningrum et al., 2016). After activation of NIH-3T3 cells with 20 ng/ml TGFβ for 48 h, equal amounts of activated NIH-3T3 and A549 cells were mixed and seeded into ultralow-attachment round-bottom culture plates with 300 rpm shaking, followed by incubation at 37 °C for 3 days. After heterospheroid formation, the spheroids were separately incubated with different formulations of L-DIL for 4 h. The heterospheroids were fixed by PFA and cleared by FocusClear (CelExplorer, Taiwan). After nuclear staining with SYTO16 dye, the spheroids were carefully transferred to cambered coverslips and scanned from the top. Each image was measured by CLSM Z-stack scanning (pinhole: 1.7 µm; Z interval: 1.0 µm between consecutive slides) using a JelloX Biotech system.

To digitize the Z-stack images of each spheroid, the intensity data of each image were calculated using Avizo^®^ software for quantitative analysis. The stack images of each spheroid were analyzed as a volumetric sphere using segmentation of the nucleus signal. After finding the BaryCenter of each sphere, we calculated the nucleus size with nucleus signal using H-maxima and watershed algorithms. Dil signal analysis by distance was performed by single seeded distance mapping from the BaryCenter of each volumetric sphere mentioned above.

### Establishment of human lung adenocarcinoma (ADC) xenograft mouse model

Six-week-old female C.B.17/Prkdc^scid^/CrlNarl mice (18–20 g) were obtained from the National Laboratory Animal Center (Taiwan), and housed at 20 ± 1 °C with access to food and water ad libitum in a specific pathogen-free (SPF) environment. All animal experiments were carried out in accordance with protocols evaluated and approved by the animal ethics committee of NTHU. A549-iRFP cells (*1 × 10^6^*) suspended in 50% Matrigel (BD Biosciences, Germany)/PBS solution (1:1, v/v), and implanted subcutaneously in the lateral flank of each mouse. After the tumor volume of mice grew up to around 100–200 mm^3^, the mice were allowed to undergo intravenous administration for distribution assay and therapeutic effect.

### Doxorubicin quantification in tissue for *in vivo* distribution

Doxorubicin was extracted from tissues as described (Alhareth et al., 2012). Tissues including heart, liver, spleen, lung, tumor, and muscle were harvested and homogenized. Homogenized tissue dissolved in 0.1 M SDS was spiked with equivalent 20 μg/ml idarubicin (internal standard). After adding equal 1 M Tris buffer (pH 8.5), extraction of anthracyclines was mixed by chloroform/methanol (9:1, v/v). After stirring for 3 min, the samples were centrifuged at 1000 ×*g* RT for 5 min, and the organic phase was collected and evaporated to dryness at 30 °C. For quantification, the samples were analyzed by HPLC. The mobile phase consisted of a mixture of 0.05 M trichloro-acetic acid and acetonitrile (70/30, v/v). Detection of doxorubicin was performed with UV detector at wavelength of 450–500 nm.

### *In vivo* therapeutic effect

A549-iRFP tumor-bearing mice whose solid tumor reached a volume of 100 mm^3^ were randomly divided into six groups (6 mice per group). Mouse was intravenously administrated *via* tail vein with the above formulations at a dose of 2 mg/kg of doxorubicin per week for four weeks, meanwhile body weight and tumor volume were recorded during the experiment. Tumorigenic capacity was calculated as the following formula: V = (L × W^2^)/2, in which L is tumor dimension of the longest point, and W is that of the widest point. Tumor volume growth and weight change were graphed to reveal the trends upon drug treatment. In addition, the mice were sacrificed by CO_2_ narcosis. Finally, tumor of these mice was weighted and fixed with 4% paraformaldehyde for frozen sections.

### *In vivo* imaging

The mice were anesthetized by 3% isoflurane oxygen, and immediately imaged in fluorescence channels with up to 10 sec acquisition time. Regions of interest were drawn over the tumor area for each image. Filter channels were the following: 675/20 nm exciter and 720/20 nm emitter for iRFP. All quantitative measurements of fluorescence signals as well as their linear spectral unmixing were performed using the Living Image v. 4.3.1 software.

### Immunofluorescence staining

Tissue samples were snap frozen in OCT and stored at −80 °C until processing. Tissue sections were fixed in 4% paraformaldehyde at 4 °C for 10 min and incubated in blocking buffer at room temperature for 1 h, and subsequently incubated with primary antibody at 4 °C overnight. After washing, the samples were then incubated with the secondary antibodies at 37 °C for 1 h. The sections were then washed in PBST and then mounted, and imaged using a fluorescence microscope (Zeiss Cell Observer-Z1, Germany).

### Statistical analysis

Statistical analysis was undertaken using Prism 5.0c Software. All experiments were at least three-time repetition, and data was presented as mean ± standard deviation. The statistical significance in mean values was determined by two-tail student’s *t*-test. Asterisks show level of statistical significance: *p < .05; **p < .01; ***p < .001 in comparison with control.

## Results

### Design and preparation of HS-targeted GBP-modified NPs

To improve the efficiency of NP targeting to tumors, we adopted a ligand-mediated HS-targeting strategy using HS-dependent GBP to generate GBP-modified liposomal drugs *via* the post-insertion method. First, DSPE-PEG_2k_-maleimide was specifically conjugated by a Michael addition reaction to the thiol group of cysteine located at the sixth position of GBP. To obtain high-quality DSPE-PEG_2k_-GBP, HPLC was used to separate the raw materials as well as our product, DSPE-PEG_2k_-GBP (Supplementary Figure S2). As shown in Supplementary Figure S3, the mixture was separated using the indicated program. An additional peak at a retention time of 24.05 min was observed only on chromatograms of the DSPE-PEG_2k_-GBP mixture, indicating the formation of the final product. As expected, the mass spectra of DSPE-PEG_2k_-GBP show signals ranging from 1600–2600 m/z to 3900–5000 m/z with a major peak at 4342.6 m/z, evidently indicating the successful synthesis of DSPE-PEG_2k-_GBP.

DSPE-PEG_2k_-GBP was incorporated into L-DOX by a post-insertion method to develop a novel formulation of GBP-modified L-DOX (L-DOX-GBP) with different molar percentage (mol%) modifications (Allen et al., 2002). The L-DOX formulations were generated with different GBP densities, and 1 mol%, 2 mol% and 3 mol% GBP modifications were labeled as L-DOX-GBP_(L)_, L-DOX-GBP_(M)_, and L-DOX-GBP_(H)_, respectively. In addition, the 3 mol% methoxy-PEG-modified L-DOX (L-DOX-PEG_(H)_) group was tested to rule out the possibility of efficiency enhancement due to simply introducing pegylation modification on the liposomes.

### NP property characterization of L-DOX-GBP

The size and shape characterization of L-DOX were carried out by negative stain transmission electron microscopy (TEM), a powerful technique for investigating morphology of macromolecular structures and complexes. From the TEM images in Supplementary Figure S4(A), different formulations of L-DOX displayed a uniform spherical shape with an average size of about 80–140 nm, even though some bilayer liposomal membrane collapsed due to dryness procedure. Such particle size distribution was consistent with characterization by dynamic light scattering (Supplementary Figure S4(B)). The physical properties are summarized in Table 1. The mean particle size and zeta potential of L-DOX were determined to be 121.6 ± 0.8 nm and −9.69 ± 0.73 mV, respectively. As expected, the mean particle sizes of L-DOX-GBP_(L)_, L-DOX-GBP_(M)_, and L-DOX-GBP_(H)_ were 126.3 ± 3.7, 127.1 ± 5.3, and 128.5 ± 5.6 nm, respectively. Moreover, the zeta potentials of L-DOX-GBP_(L)_, L-DOX-GBP_(M)_, and L-DOX-GBP_(H)_ were −4.44 ± 0.01, −3.43 ± 0.33, and −2.09 ± 0.02 mV, respectively, indicating that our cationic GBP increased the amount of positively charged moieties on the liposome surface. Taken together, these data show that the incorporation of DSPE-PEG_2k_-GBP into L-DOX slightly increased the particle size within 10 nm and the cationic charge of L-DOX but did not lead to aggregation. Moreover, *in vitro* drug lease profile at 37 °C for 72 h was shown in Supplementary Figure S5. Approximately 10% drug leakage in all groups was observed within 12 h probably due to incorporation process, and the leakage in all formulations did not show significant difference within 72 h incubation. These formulations of L-DOX-GBP were adopted for further evaluation of *in vitro* and *in vivo* functions.

**Table 1.** Particle diameter and zeta potential of peptide-modified liposomal drugs  .

	L-DOX	L-DOX-PEG_(H)_	L-DOX-GBP_(L)_	L-DOX-GBP_(M)_	L-DOX-GBP_(H)_
Size (nm)	121.6 ± 0.8	130.0 ± 6.6	126.3 ± 3.7	127.1 ± 5.3	128.5 ± 5.6
Zeta potential (mV)	−9.69 ± 0.73	−11.34 ± 0.71	−4.44 ± 0.01	−3.43 ± 0.33	−2.09 ± 0.02

### HS-dependent cellular uptake

**Figure 1.**
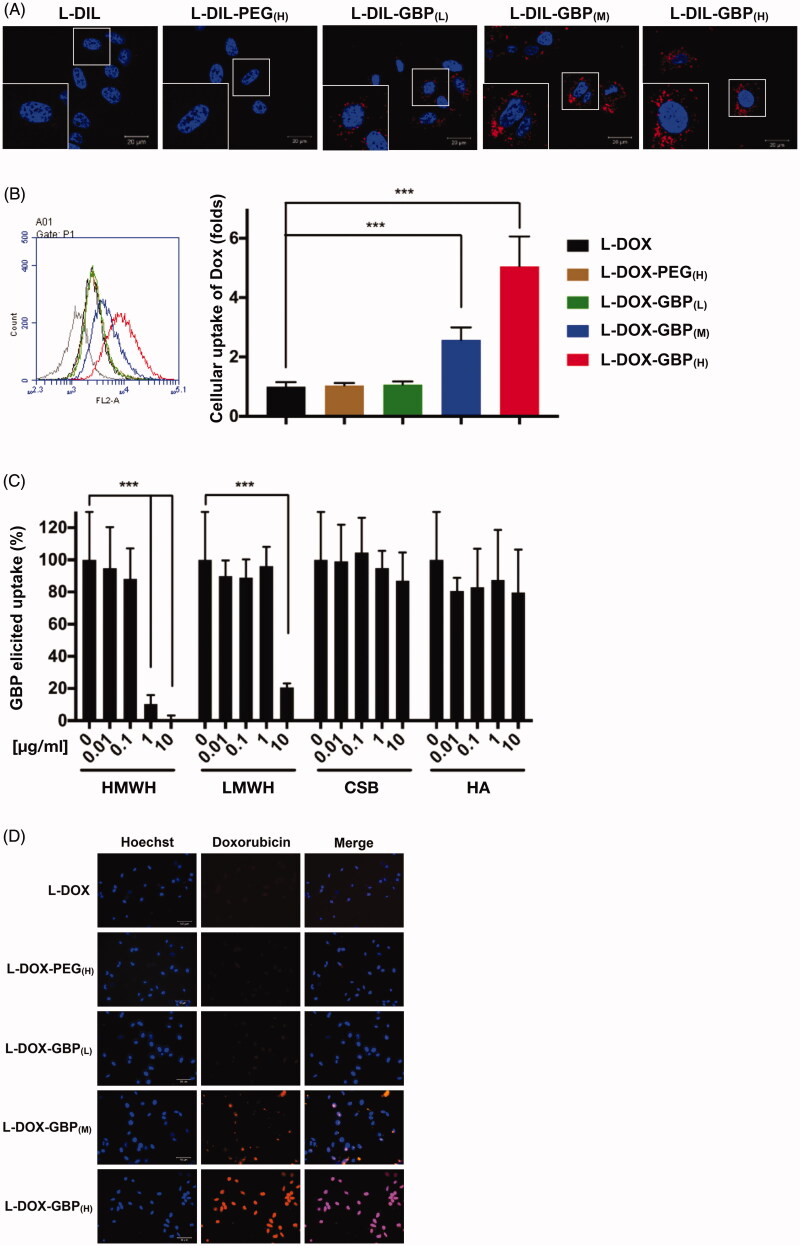
HS-dependent cellular uptake of GBP-modified pegylated liposomes. (A) The A549 cells were incubated with DiI-labeled liposomes (L-DIL) and GBP-modified L-DIL (L-DIL-GBP) at 37 °C for 4 h and were monitored by confocal microscopy. (B) The cells were incubated with L-DOX and L-DOX-GBP at 37 °C for 24 h, and cellular uptake and drug release were assessed by flow cytometry. (C) The cells were treated for 24 h with L-DOX-GBP_(H)_ in the presence of the indicated concentrations of GAGs, including high-molecular-weight heparin (HMWH), low-molecular-weight heparin (LMWH), chondroitin sulfate type B (CS), and hyaluronic acid (HA), and the cellular uptake of doxorubicin was analyzed. The cellular uptake of the L-DOX treatment group was used to normalize that of the L-DOX-GBP(H) group upon GAG competition. (D) The cells were separately treated with 10 μM L-DOX and L-DOX-GBP at 37 °C for 24 h, and cellular uptake and drug release were assessed by fluorescence microscopy. Magnification: 40×; scale bar: 50 μm. The data are the mean ± SD, averaged from three separate experiments. *p < .05; **p < .01, two-tailed Student’s *t*-test.

Efficient cellular uptake of NP by endocytosis is a prerequisite for drug delivery. Even if doxorubicin is naturally fluorescent, doxorubicin self-quenches in liposomes until drug release. To trace and visualize the liposomes, DiI, a lipophilic tracer, was used to stain the liposomes to generate GBP-modified L-DIL (L-DIL-GBP). The cellular uptake of L-DIL-GBP was subsequently monitored by confocal microscopy. The signals of DiI were barely observed in the membrane and cytosol of A549 cells, revealing that L-DIL and L-DIL-PEG_(H)_ were not able to directly fuze with or penetrate across the plasma membrane (Figure 1(A)). On the other hand, punctate cytosolic DiI signals were evidently detected in the L-DIL-GBP_(L)_, L-DIL-GBP_(M)_, and L-DIL-GBP_(H)_ treatment groups, indicating that L-DIL-GBP successfully penetrated into the A549 cells through the endocytic pathway but did not undergo direct translocation. Subsequently, the therapeutic drugs L-DOX and L-DOX-PEG_(H)_ and three formulations of L-DOX-GBP were separately incubated with A549 cells to monitor internalization and release by flow cytometry. As shown in Figure 1(B), the cellular uptake of L-DOX-PEG_(H)_, L-DOX-GBP_(L)_, L-DOX-GBP_(M)_, and L-DOX-GBP_(H)_ was respectively 1.0 ± 0.1-, 1.1 ± 0.1-, 2.6 ± 0.4-, and 5.7 ± 0.2-fold higher than that of L-DOX, but not in normal bronchus cell line (Supplementary Figure S6). Thus, GBP modification significantly enhanced the L-DOX uptake of A549 cells.

We continued to test whether the interaction between HS and GBP is critical for the cellular uptake of L-DOX-GBP. Here, two types of HS analogs (LMWH and HMWH), CS, and HA, were tested for competition. Enhanced internalization of L-DOX-GBP_(H)_ was significantly abolished in the presence of 1 and 10 μg/ml HMWH as well as 10 μg/ml LMWH, but not in the presence of CS and HA (Figure 1(C)). As the GBP-induced L-DOX uptake was blocked only by exogenous HS, these data suggested that HS serves as the key for the internalization of L-DOX-GBP.

### *In vitro* cytotoxicity of L-DOX-GBP

Peptide-functionalized NPs, such as trans-activating transcription (TAT) peptide modification, induce a multivalent effect, which induces clathrin-mediated endocytosis *via* strong interaction with the cell through multiple receptors (Bartczak et al., 2012; Oh & Park, 2014; Dalal & Jana, 2017). This multivalent effect might cause dynamic endocytosis/exocytosis, restricting subcellular performance and drug release of NPs. We found that the signal of doxorubicin, which is a fluorescent DNA chelator, was highly overlapped with that of nuclear staining in the L-DOX-GBP_(M)_ and L-DOX-GBP_(H)_ treatment groups (Figure 1(D)), suggesting that doxorubicin was indeed released from the liposome and accumulated in the cell nucleus. Cell viability upon treatment with L-DOX slightly declined as the drug concentration increased from 2.5 to 40 μM, and the IC_50_ of L-DOX was calculated to be 13.1 ± 3.1 µM (Table 2). As expected, the viability of A549 cells upon treatment with L-DOX-GBP_(M)_ and L-DOX-GBP_(H)_ significantly declined compared with that of the L-DOX group, and the IC_50_ values of L-DOX-GBP_(M)_ and L-DOX-GBP_(H)_ were calculated to be 8.3 ± 1.5 and 5.7 ± 1.0 µM, respectively, while the IC_50_ value of L-DOX-GBP_(L)_ treatment was 9.0 ± 2.9 µM. These data suggested that GBP-induced L-DOX uptake was able to enhance A549 cell death.

**Table 2.** IC_50_ of L-DOX-GBP.

Treatment	L-DOX	L-DOX-PEG_(H)_	L-DOX-GBP_(L)_	L-DOX-GBP_(M)_	L-DOX-GBP_(H)_
IC_50_ (µM)	13.1 ± 3.1	19.4 ± 5.4	9.0 ± 2.9	8.3 ± 1.5	5.7 ± 1.0

Cells were incubated with with LipoDox and L-DOX-GBP at doxorubicin concentration of 0 to 40 µM for 24 h followed by the incubation period of 48 h before cell viability was measured.

### Drug penetration in tumor-stroma-containing heterospheroids

**Figure 2.**
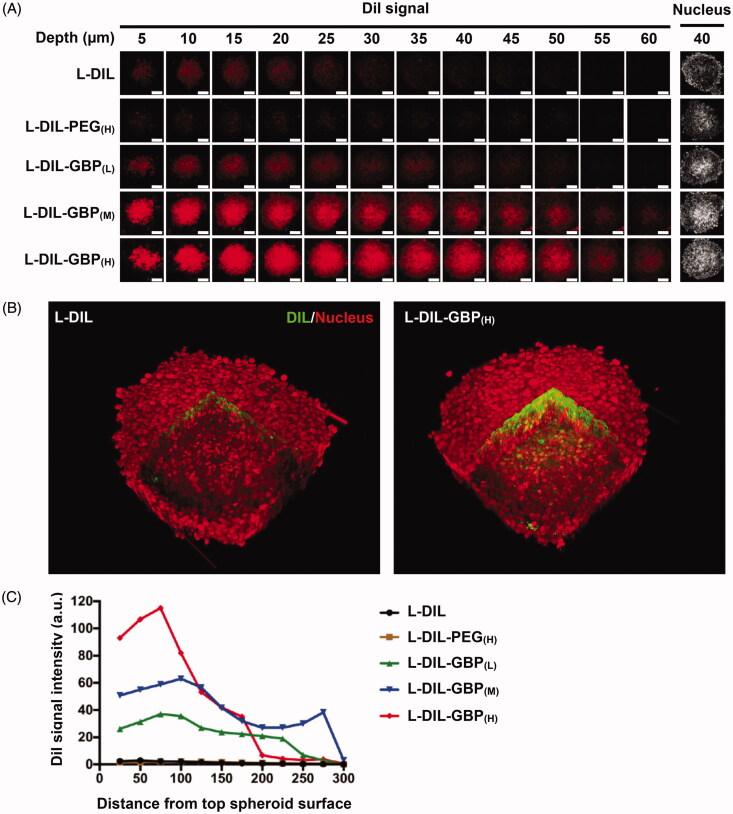
Drug penetration activity of L-DIL-GBP in spheroids. Heterospheroids composed of A549 and NIH-3T3 cells were incubated with different formulations of L-DIL for 4 h. (A) Penetration capacity was measured by CLSM Z-stack scanning with pinhole: 1.7 μm; Z interval: 1.0 μm between consecutive slides. Nuclei stained by SYTO16; DiI (red). Magnification: 20×; scale bar: 100 μm. (B) Three-dimensional images were reconstructed to illustrate L-DIL or L-DIL-GBP(H) penetration into the heterospheroids. DiI signal (green); nucleus (red). (C) Quantitative analysis between the mean intensity of the Dil signal and the distance to the center of the spheroid. The data are the mean ± SD, averaged from three separate experiments. *, **, and *** indicate *p* < .05, *p* < .01 and *p* < .001 under the two-tailed *t*-test, respectively.

We intended to explore whether GBP modification enhanced penetration efficiency into spheroids. Crowded tumor stroma causes poor tissue penetration of NP, and 3D tumor heterospheroids provide a powerful platform for studying tumor biology because their morphology and microenvironment resemble that of solid tumors with a desmoplastic architecture phenotype (Priwitaningrum et al., 2016). In Figure 2(A), the consecutive CLSM Z-stack scanning images show that the DiI signals of the L-DIL and L-DIL-PEG_(H)_ treatments were mostly located at the periphery of the spheroid (a depth of 5–25 µm) and were rarely detected in the interior area of the spheroid deeper than 30 µm, suggesting that the heterospheroids blocked NP penetration. The L-DIL-GBP treatments demonstrated a deeper penetrating degree than did the L-DIL and L-DIL-PEG_(H)_ treatments. Importantly, the reconstructed three-dimensional images illustrate the extensive location of L-DIL with 3 mol% GBP modification (Figure 2(B)). Quantitative analysis between the mean intensity of the L-DIL signal and the distance to the center of the heterospheroid was performed. A high density of GBP modification increased L-DIL accumulation to a depth of 100 μm from the spheroid surface (Figure 2(C)). Consistent with the 2D *in vitro* internalization study, GBP modification facilitated L-DIL across the pathological barrier to penetrate into the inner space of the spheroid, especially in the cases of the L-DIL-GBP_(M)_ and L-DIL-GBP_(H)_ treatments.

### *In vivo* tumor accumulation and tissue penetration of L-DOX-GBP

**Figure 3.**
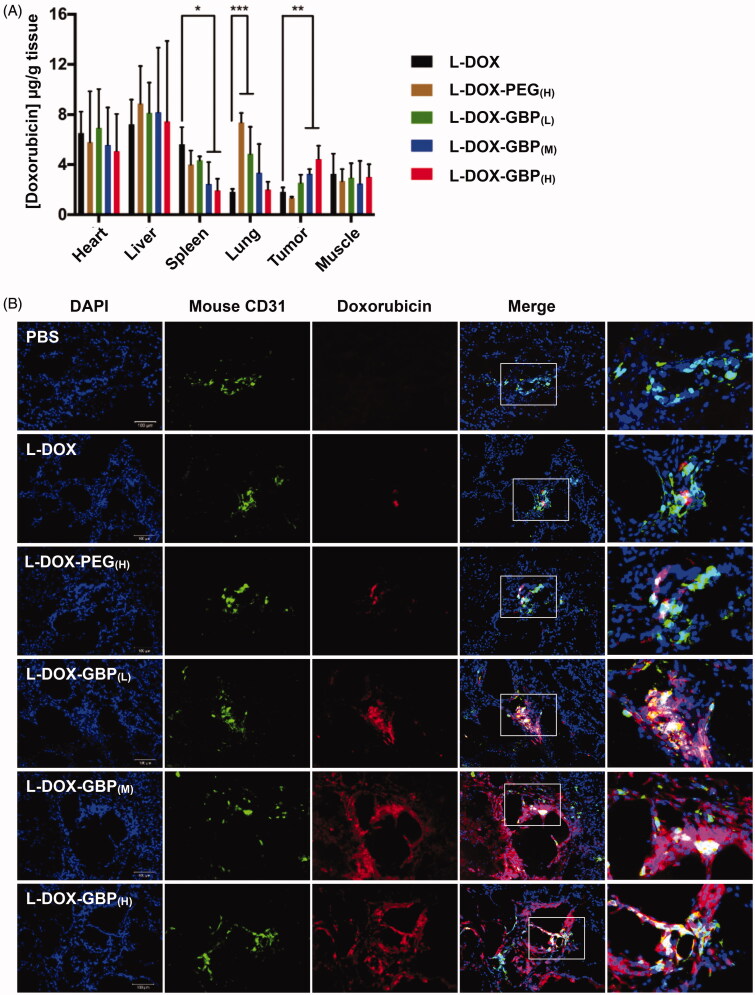
Drug accumulation and tumor tissue penetration of L-DOX-GBP. A549-iRFP tumor xenograft mice were intravenously injected with different formulations of L-DOX at 2 mg/kg concentration of doxorubicin, and the tissues were harvested 4 days after administration (*n* = 3). (A) Doxorubicin accumulation in the heart, liver, spleen, lung, tumor, and muscle was quantified by HPLC. (B) The tumor tissues were stained with an angiogenesis marker using an anti-CD31 antibody. The endothelium (green), nuclei (blue), and doxorubicin signals (red) were monitored. Magnification: 20×; scale bar: 100 μm.

The heart, liver, spleen, lung, muscle, and tumor tissues were harvested and homogenized for doxorubicin quantification by HPLC to evaluate the *in vivo* targeting activity of L-DOX, L-DOX-PEG, and L-DOX-GBP. Figure 3(A) summarizes the doxorubicin levels in tissues following a single injection of five different doxorubicin formulations in A549-tumor-bearing mice. A significantly high doxorubicin content was detected in the tumors of the L-DOX-GBP_(M)_ (1.9-fold) and L-DOX-GBP_(H)_ (2.6-fold) groups compared with that of the L-DOX treatment group, revealing that 2 mol% and 3 mol% GBP modifications on the L-DOX surface facilitated drug accumulation in the tumors. As expected, the highest concentration of doxorubicin accumulated in the liver in all treatments (7–9 µg/g tissue) due to major clearance from the circulation by the liver, suggesting that additional pegylation or GBP modification on L-DOX did not influence hepatic clearance. Likewise, doxorubicin levels in the heart and muscle were similar in all treatments. Surprisingly, the doxorubicin contents in the spleen of the L-DOX-GBP_(M)_ (42%) and L-DOX-GBP_(H)_ (34%) groups were low compared with that of the L-DOX treatment group, suggesting that GBP modification might minimize splenic clearance. In addition, the doxorubicin concentration in the lung tissue of the L-DOX-PEG_(H)_ and L-DOX-GBP_(L)_ groups increased 4.1- and 3.3-fold, respectively, compared to that of L-DOX, while similar contents as that of the L-DOX treatment group were observed in the L-DOX-GBP_(M)_ and L-DOX-GBP_(H)_ groups.

Subsequently, immunofluorescence staining was performed to confirm L-DOX accumulation in the tumor tissues to further investigate drug distribution. Consistent with the results of doxorubicin accumulation (Figure 3(A)), very little doxorubicin signal was released and observed in tumor tissues of the L-DOX and L-DOX-PEG_(H)_ treatment groups (Figure 3(B)), presumably due to a lack of active targeting efficiency. Moreover, those signals still remained in the nearby endothelium, revealing that L-DOX and L-DOX-PEG_(H)_ failed to overcome the interstitial space barrier to penetrate deeply into the tumor tissues. Interestingly, drug escape from the barrier was observed in the L-DOX-GBP_(M)_ and L-DOX-GBP_(H)_ groups, strongly indicating that surface modification with GBP led to extensive drug distribution of L-DOX in tumor tissue. Notably, although drug accumulation seemed to increase in the L-DOX-GBP_(L)_ group (Figure 3(B)), the doxorubicin signals mainly surrounded the blood vessels. These data suggested that GBP modification facilitated not only L-DOX extravasation into tissues but also its escape from tumor vessels.

### *In vivo* anti-tumor efficiency of L-DOX-GBP

To evaluate anti-tumor efficiency, low-dose therapeutic treatment with L-DOX-GBP was applied to the A549-iRFP tumor-bearing mouse model. The mice were treated separately with PBS, L-DOX, L-DOX-PEG, and L-DOX-GBP by intravenous administration of 2 mg/kg doxorubicin once a week for 4 weeks and sacrificed for further investigation. After 4 weeks of therapeutic treatment, the tumor volumes of the tumor-bearing mice treated with PBS, L-DOX, L-DOX-PEG_(H)_, L-DOX-GBP_(L)_, L-DOX-GBP_(M)_ and L-DOX-GBP_(H)_ grew to 638.8 ± 137.89, 428.2 ± 55.71, 400.1 ± 84.81, 349.6 ± 105.16, 260.8 ± 66.02 and 201.0 ± 66.92 mm^3^, respectively (Figure 4(A)), clearly revealing that the degrees of tumor growth inhibition were 39.1%, 44.3%, 53.7%, 70.2%, and 81.3%, respectively, in comparison with that of the PBS treatment. To better monitor tumor growth upon drug treatment, the IVIS system was used for quantitative analysis of the A549-iRFP cells. Figure 4(B,C) illustrates that the photon counts were 16.6 ± 4.67 × 10^6^, 11.1 ± 2.76 × 10^6^, 10.2 ± 4.62 × 10^6^, 7.9 ± 2.08 × 10^6^, 6.5 ± 3.40 × 10^6^, and 5.1 ± 2.07 × 10^6^ after 4 weeks of administration, and the inhibition rates were calculated to be 33.0%, 38.8%, 52.7%, 61.1%, and 69.2% compared with that of the PBS group, indicating that tumor growth significantly plummeted in the human xenograft lung ADC model upon treatment with L-DOX-GBP_(M)_ and L-DOX-GBP_(H)_. In addition, the relative body weight ratios (W_a_/W_0_) of the mice were measured to be 103.1 ± 9.28%, 88.1±.9.69%, 107.6 ± 4.09%, 99.3 ± 1.41%, 103.0 ± 8.19%, and 99.25 ± 6.14% after treatment (Figure 4(D)). The body weight reduction of the L-DOX treatment group significantly declined in comparison with those of the L-DOX-PEG_(H)_ group and other formulations at the end of the experiment. Solid tumors were harvested and weighed to be 0.87 ± 0.185, 0.57 ± 0.115, 0.48 ± 0.098, 0.52 ± 0.216, 0.37 ± 0.097, and 0.32 ± 0.162 g (Figure 4(E)). These data demonstrated that L-DOX slightly inhibited A549 cell growth; moreover, L-DOX-GBP_(M)_ and L-DOX-GBP_(H)_ exhibited a better anti-tumor effect than did L-DOX, L-DOX-PEG_(H)_, and L-DOX-GBP_(L)_. As expected, doxorubicin still encircled the vessels even after a four-dose course of therapy upon treatment with L-DOX and L-DOX-PEG_(H)_ (Figure 4(F)). In addition, doxorubicin accumulated extensively in the tumor tissues and spread from the blood vessels to the depths of the tumor tissues, revealing that GBP modification indeed enhanced therapeutic efficiency and improved distribution.

Figure 4.*In vivo* therapeutic efficacy of L-DOX-GBP in A549-iRFP tumor xenograft. A549-iRFP tumor xenograft mice were intravenously injected with different formulations of L-DOX at 2 mg/kg concentration of doxorubicin per week for 4 weeks. (A) The tumor volume was monitored and calculated twice per week during the 4-week therapeutic period (*n* = 6). (B) A live imaging system was used to confirm tumor size and (C) quantify the iRFP signals of A549-iRFP cells. (D) Body weight change upon therapeutic treatment was monitored. (E) The solid tumor was weighed after scarification. (F) A549-iRFP tumor tissues after therapeutic treatment were stained with anti-CD31 antibody. The endothelialcells (green), nuclei (blue), and doxorubicin signals (red) were monitored. *, **, and *** indicate *p* < .05, *p* < .01 and *p* < .001 under the two-tailed *t*-test, respectively. Magnification: 20×; scale bar: 100 μm.

**Figure:**
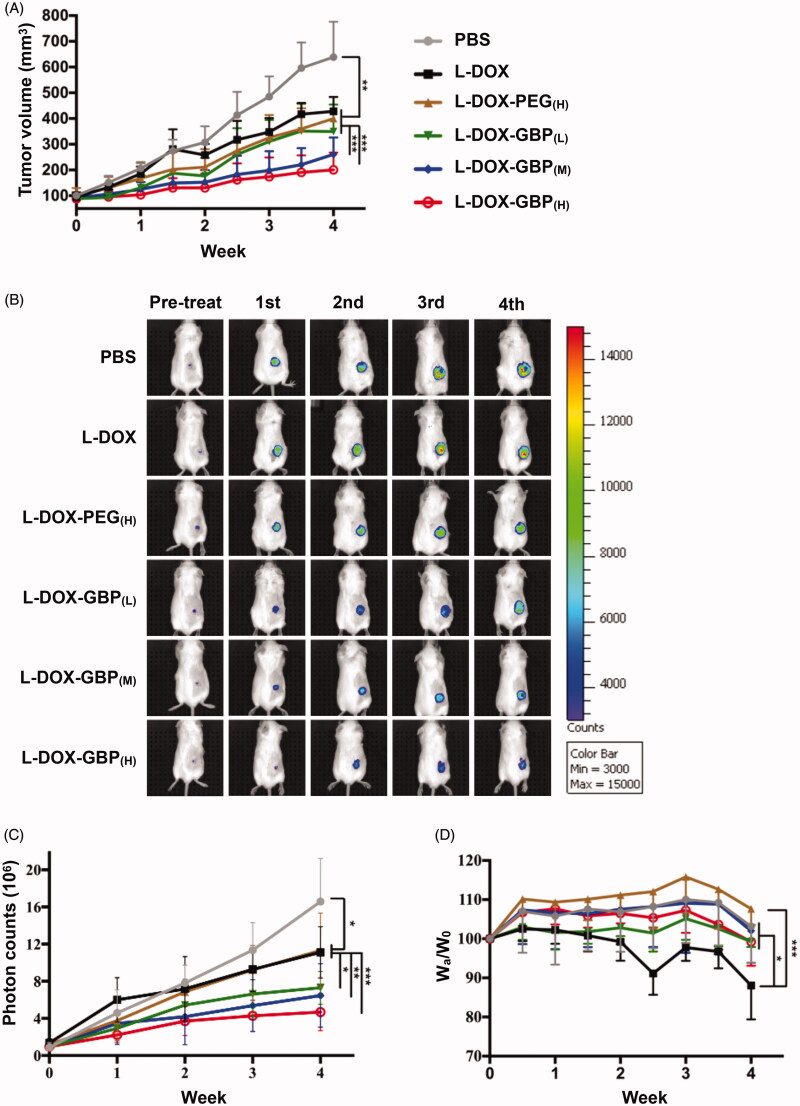

**Figure:**
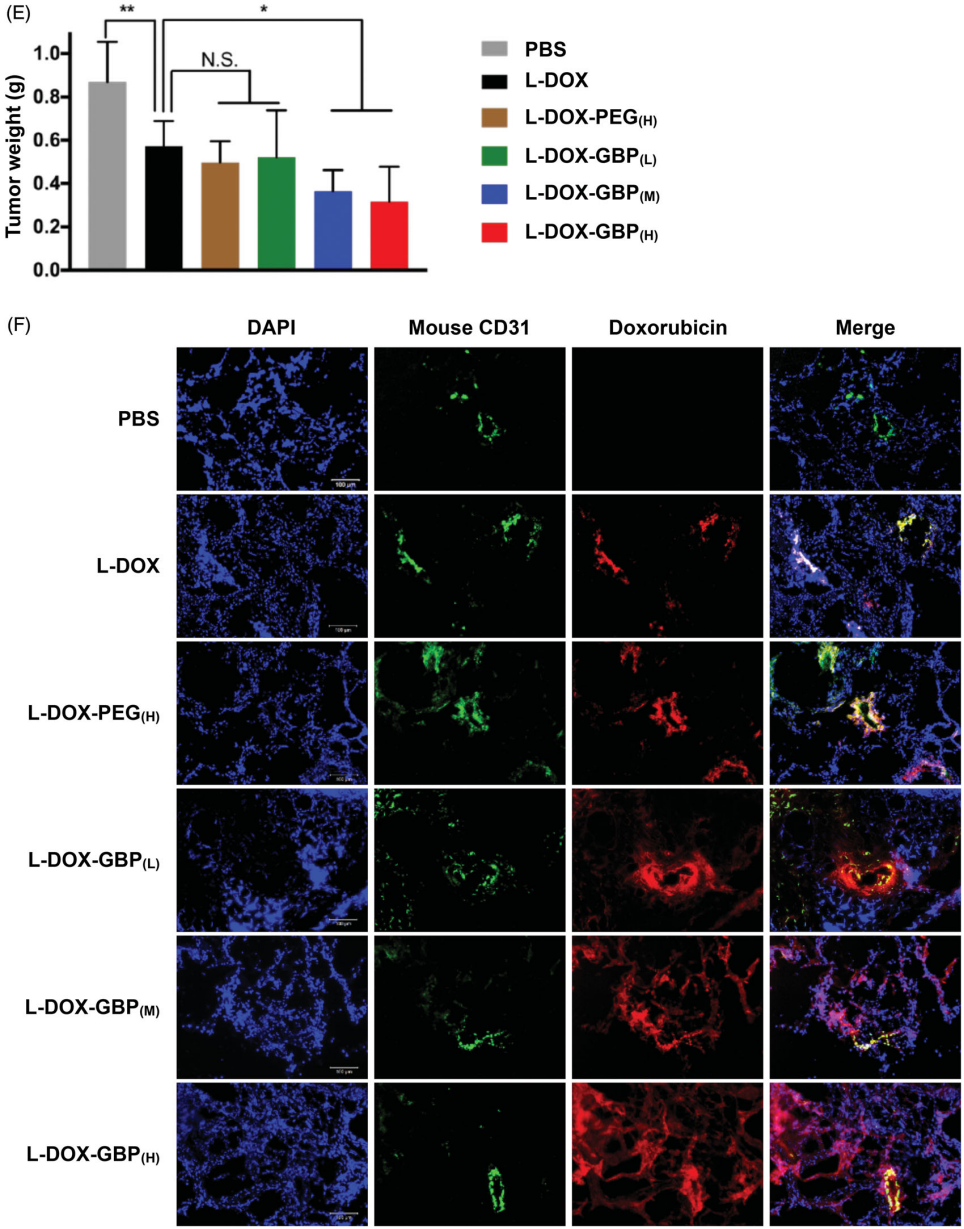

## Discussion

Intratumoral delivery of NPs requires several steps in transport, including vascular transport, extravasation, interstitial transport, and cellular uptake, which are generally limited by the pathophysiological properties of tumors. Several studies have been dedicated to developing advanced drug delivery systems (Yang & Gao, 2017). Based on those characteristics and the aims of targeting and penetrating tumor tissues, pH-sensitive, enzyme-triggered, temperature-sensitive, or light-responsive NP-based drug delivery systems have been developed (Hu et al., 2018; Liu et al., 2019). Here, peptide-NP conjugates that actively target different components of the tumor stroma to enhance NP extravasation and penetration into tumor tissues. For instance, iRGD, the most famous tumor targeting and penetrating peptide, successfully homes NPs to the tumor vasculature by the RGD motif and spreads into tumor tissue by the CendR motif (Sugahara et al., 2009; Teesalu et al., 2009).

ECM serves as the major physical barrier of extravasation and penetration of biomacromolecules. The strategy of targeting neoplasm ECM increases NP accumulation in tumors to enhance anti-tumor efficiency. Nevertheless, interacting with the ECM might impede NP diffusion and inhibit penetration (Stylianopoulos et al., 2010). Recently, a fibronectin-targeting NP demonstrated enhanced retention and tumor cell uptake in spheroids and *in vivo*, leading to a deep distribution in the tumor tissue (Zhang et al., 2014). Moreover, a dual ECM targeting strategy using an anti-tenascin-C aptamer and cationic CPP demonstrated that cationic CPP might facilitate NP movement in tumor tissue (He et al., 2018). Here, abnormally abundant HS and HSPGs in the tumor ECM served as a target for liposomal NP with surface modification by GBP. L-DiI blocked the outside of the spheroid; however, 2 to 3 mol% GBP modification facilitated penetration into the interior of the spheroid (Figure 2), presumably because HS attracted the L-DIL-GBP into the core of the heterospheroids and functioned as an anchor to enhance the retention after removal of the excess L-DIL *in vitro* (Zhang et al., 2014). Moreover, L-DOX-GBP demonstrated enhanced accumulation and much extensive distribution on A549 tumor tissues compared with that of conventional L-DOX *in vivo*. HS and HSPGs around the neoendothelium were capable of promoting L-DOX-GBP extravasation from vessels and form co-aggregates, enhancing L-DOX-GBP retention in tumor tissue (Zhang et al., 2014; He et al., 2018). However, we did not provide direct evidence to explain the extensive distribution of L-DOX-GBP across the interstitial space. This phenomenon might be associated with the ligand diffusion characteristic of HS. FGF- and FGF-labeled gold NPs diffused through HS chains of the peri-cellular matrix and were finally transported in the interstitial space, thus enhancing cellular uptake (Duchesne et al., 2012; Sun et al., 2016).

Components of the mononuclear phagocytic system (MPS), macrophages in particular, are key cellular participants in detecting exogenous NP and initial clearance, representing major barriers for the implementation of clinical use of NPs (Hoshyar et al., 2016). MPS of liver Kupffer cells and splenic marginal zone (MZ) macrophages serve as key players in the clearance of exogenous NPs (10–200 nm). The hepatic clearance was not significantly different with or without GBP modification, presumably due to constant size properties in the different formulations (Tsoi et al., 2016). Surprisingly, splenic uptake was significantly reduced in the 2 mol% and 3 mol% GBP modification groups. Splenic macrophages not only capture NPs by recognizing surface chemistry but are also involved in the adaptive immune system by accelerated blood clearance (ABC) phenomenon toward pegylated NPs (Demoy et al., 1999; Ishida et al., 2006; Verhoef & Anchordoquy, 2013). Surface modification agents, such as the CD47 fragment peptide, inhibited phagocytic clearance of NPs by a ‘self’ signal, revealing that the surface modified NPs successfully influenced macrophage recognition and activation (Rodriguez et al., 2013). Our GBP reported decreased macrophage recruitment and cytokine secretion *in vivo* (Fu et al., 2017), suggesting that this peptide possessed immunomodulatory activity. These properties might contribute to the escape of the pegylated L-DOX surface from splenic uptake. L-DOX that underwent additional pegylation modification became trapped in capillaries in the lung, and a high-percentage GBP modification alleviated this accumulation. Taken together, these results showed that HS targeting by GBP modification might slightly influence the distribution and clearance of L-DOX.

Recently, more and more studies suggested that formation of protein corona on NP surface might be another emerging issue for NP delivery. As soon as entry to the blood, the absorption of protein onto the NP surface intervene cellular uptake *in vitro*, recognition by reticuloendothelial system, and distribution *in vivo* (Aggarwal et al., 2009; Corbo et al., 2017). Composition, thickness, and decoration of protein corona are relied on assorted factors including physicochemical properties and environmental proteins (Bigdeli et al., 2016; Saha et al., 2016; Xiao et al., 2018). Several studies further revealed that absorption of protein could lead to off-target effect of ligand functionalized NPs (Sacchetti et al., 2013; Xu et al., 2016). For example, Mohamadreza Amin et al. reported that TAT-modified liposome obviously increased *in vitro* cellular uptake, reduced splenic clearance, and improved *in vivo* distribution and therapeutic efficiency (Amin et al., 2018). The increased thickness of protein corona shields TAT cationic properties of liposome, and the presence of TAT moieties on NP surface might result in alteration of protein absorptions (such as inhibiting opsonins adsorption), contributing to perform the stealth-like behavior during circulation. Moreover, the influence on cellular uptake of NPs *in vivo* might be further evaluated in exposure of serum or other extracellular proteins. Protein corona formation limited the ligand-mediated translocation performance *in vitro*, however, the neutralization phenomenon did not work *in vivo* (Amin et al., 2018; Zhang et al., 2018). Surprisingly, ligand functionalized NPs seemed to conserve the ligand moieties in spite of absorption of protein onto their surface *in vivo* (Amin et al., 2018). In fact, the composition of protein corona was influenced by different protein source, concentration, or incubation condition. Pozzi et al. demonstrated that the composition of protein corona was different under static or dynamic condition *in vitro*, suggesting that protein corona formation was dynamic process and was replaceable (Pozzi et al., 2015). The protein corona is initially composed of serum protein with high concentration and high association rate constant as entry into blood. While leaving circulation, these proteins theoretically dissociate and were replaced by other proteins from the targeted tissues at lower concentration, low exchange rate, and higher affinity to NP surface because of dramatic condition change (such as acidic pH value, protein assortments and concentration in tumor tissue) (Cedervall et al., 2007; Lynch et al., 2009; Monopoli et al., 2011). This exchange may allow functionalized NP to display ligand property, which might be an important process to reason the discrepancy between *in vitro* and *in vivo* performance for some ligand-mediated delivery  (Amin et al., 2018; Zhang et al., 2018).

In summary, the clinical usage of NP is limited by the desmoplastic stroma in tumors of NSCLC, which impedes the accumulation and penetration of NPs in tumor tissues. In this study, a novel therapeutic approach is designed in which a new agent specifically targets the tumor stroma. Specifically, an HS-targeting liposome conjugated with GBP was developed to treat human lung adenocarcinoma tumors in which a high density of ECM aggregated on the tumor desmoplastic stroma. This novel formulation of L-DOX-GBP selectively accumulated in the tumor site either *via* the EPR effect or by active targeting to the HS-rich stroma. In addition, GBP conjugation increased the spheroid and tumor penetration activity of liposomal NPs. Moreover, surface modification with GBP elicited HS-dependent cellular uptake and released drugs deep into the tumor tissue. Optimized drug distribution in the tumor site and excellent anti-tumor efficiency were observed both *in vitro* and *in vivo*. Based on the unique interaction between GBP and HS in the tumor stroma, selective HS-targeting NPs with a deep distribution may serve as a novel approach for targeting desmoplastic tumors.
